# Genome-Wide High-Throughput Screening to Investigate Essential Genes Involved in Methicillin-Resistant *Staphylococcus aureus* Sequence Type 398 Survival

**DOI:** 10.1371/journal.pone.0089018

**Published:** 2014-02-12

**Authors:** Mette T. Christiansen, Rolf S. Kaas, Roy R. Chaudhuri, Mark A. Holmes, Henrik Hasman, Frank M. Aarestrup

**Affiliations:** 1 National Food Institute, Technical University of Denmark, Kongens Lyngby, Denmark; 2 Department of Veterinary Medicine, University of Cambridge, Cambridge, England, United Kingdom; Charité-University Medicine Berlin, Germany

## Abstract

Livestock-associated methicillin-resistant *Staphylococcus aureus* (LA-MRSA) Sequence Type 398 (ST398) is an opportunistic pathogen that is able to colonize and cause disease in several animal species including humans. To better understand the adaptation, evolution, transmission and pathogenic capacity, further investigations into the importance of the different genes harboured by LA-MRSA ST398 are required. In this study we generated a genome-wide transposon mutant library in an LA-MRSA ST398 isolate to evaluate genes important for bacterial survival in laboratory and host-specific environments. The transposon mutant library consisted of approximately 1 million mutants with around 140,000 unique insertion sites and an average number of unique inserts per gene of 44.8. We identified LA-MRSA ST398 essential genes comparable to other high-throughput *S. aureus* essential gene studies. As ST398 is the most common MRSA isolated from pigs, the transposon mutant library was screened in whole porcine blood. Twenty-four genes were specifically identified as important for bacterial survival in porcine blood. Mutations in 23 of these genes resulted in attenuated bacterial fitness. Seven of the 23 genes were of unknown function, whereas 16 genes were annotated with functions predominantly related to carbon metabolism, pH shock and a variety of regulations and only indirectly to virulence factors. Mutations in one gene of unknown function resulted in a hypercompetitive mutant. Further evaluation of these genes is required to determine their specific relevance in blood survival.

## Introduction

Bacterial genomes contain between 470 to more than 9,000 different genes [1], [2], many of which have unknown function. Detailed information on the importance and function of all genes within the genome is essential to understand bacterial survival and adaptation, especially for bacteria that may change between ecological stages as colonizers and pathogens and for those that may infect multiple hosts. Homology studies and other bioinformatic analyses of bacterial genomes have enabled prediction of gene function for many genes. However, there is still a shortage of data associating gene function with uncharacterized genes and characterized genes with phenotypes [3], as well as data on the relative importance of different genes for bacterial isolates living in different niches.

Transposon mutagenesis is a high-throughput method for functional phenotypic studies that can be utilised to associate genes to phenotypes. The method has been used to generate genome-saturated mutant libraries in several bacterial genomes [4]–[12]. The approach is based on a negative selection strategy, where transposon inserts into functional genes will result in mutants with attenuated fitness, or a complete inability to survive, and subsequent recovery of only those mutants with inserts in non-essential genes. The flanking regions of the transposon inserts can be identified and the composition of mutant libraries can be compared, pre- and post selection, resulting in identification of essential genes in a defined environment.

One genotypic approach for identifying transposon insertion sites, developed by Chaudhuri *et al.* (2009), is a DNA microarray and PCR-based method called Transposon Mediated Differential Hybridization (TMDH) [8]. This approach was applied in the first comprehensive study identifying essential genes in *Staphylococcus aureus*. Another genotypic strategy is based on high-throughput sequencing. Langridge *et al.* (2009) developed a system named Transposon Directed Insertion site Sequencing (TraDIS) which uses a transposon specific primer, enabling sequencing of the genomic target region flanking the transposon insertion sites [9]. The sequencing approach has been used by Langridge *et al.* (2009), Khatiwara *et al.* (2012), Pickard *et al.* (2013) and Chaudhuri *et al.* (2013) to study essential and conditionally essential genes in *Salmonella* Typhi and *Salmonella* Typhimurium [9]–[11], [13], but has not been applied previously to study *S. aureus* or other Gram positive bacteria. Importantly, this procedure not only identifies essential genes under different environmental conditions, but also provides an estimate of the relative importance of the presence or absence of genes.

*S. aureus* is an opportunistic pathogen that normally colonizes the host asymptomatically but given the opportunity, may cause a variety of pathogenic infections [14]. Some *S. aureus* clones are more successful human pathogens than others, and some show a high degree of host specificity for different animal species [15], [16]. Recently, a specific linage belonging to clonal complex 398 (CC398), most likely of human origin, has spread among livestock globally, acquired methicillin resistance and is now transferring back to humans leading to both colonization and disease [17]. Pigs constitute a large reservoir for livestock-associated methicillin-resistant *S. aureus* (LA-MRSA) CC398 and contribute to an ongoing spread and genetic adaptation. Comparative genomic studies have identified a few phage associated genes that appear to be correlated with virulence in humans, but no genes of importance for successful colonization or infection in livestock or other animals have been identified [18]. A greater understanding of the pathogenicity and transmission of CC398 requires further investigations into the survival mechanisms utilized by this lineage.

The aim of this study was to generate a high complexity transposon mutant library and assess the application of TraDIS in *S. aureus* Sequence Type 398 (ST398), belonging to CC398. The generated transposon mutant library was screened in laboratory and host specific environments in order to identify genes essential for ST398 to survive under the given conditions. Even though ST398 is mainly associated with pig colonization and skin infections [19], [20], *S. aureus* has potential to cause bacteraemia in pigs as well as in humans [15]. In this study whole porcine blood was applied for evaluation of the method.

## Materials and Methods

### Bacterial strains and culture conditions

The whole genome sequenced wild type (WT) livestock-associated methicillin-resistant *S. aureus* ST398 (Genbank accession AM990992) [21] and *S. aureus* RN4220 were grown in Brain Heart Infusion (BHI) (Oxoid, Difco) broth at 37°C with aeration. *S. aureus* SH1000 pMARGH2b, *S. aureus* SH1000 pFA545 and *S. aureus* RN4220 pFA545gen were grown in BHI or Tryptic Soy Broth (TSB) (Oxoid) with 5 mg/l erythromycin (Sigma), 5 mg/l tetracycline (Sigma) and 16 mg/l gentamicin (Sigma) respectively, at 30°C with aeration. For solid growth BHI agar, sheep blood agar plates (Oxoid) or Tryptic Soy Agar (TSA) (Oxoid) were applied and supplemented with the appropriate antibiotic if needed. *Escherichia coli* DH10 was cultured in Luria Broth (LB) at 37°C with aeration or on LB agar plates (Sigma).

### Plasmids

The plasmids pMARGK2b and pFA545 previously described by Chaudhuri *et al.* (2009) were used for generating a transposon mutant library in the whole genome sequenced LA-MRSA ST398 S0385 isolate. The pMARGK2b plasmid contains a mariner transposon which includes an erythromycin resistance selection marker. The plasmid backbone holds a chloramphenicol resistance selection marker and a temperature-sensitive origin replication (replication at ≤30°C). The pFA545 encodes a transposase, a temperature-sensitive origin of replication (replication at ≤30°C) and a tetracycline resistance selection marker [8]. As the LA-MRSA ST398 S0385 isolate displays natural tetracycline resistance the pFA545 plasmid was purified (Qiagen tip100) and modified. Forward primer *KpnI* and reverse primer *SpeI* (see Table 1) were used for amplification of the AAC6′-APH2′ gene encoding gentamicin resistance from MRSA MU50 DNA, The PCR product and the original pFA545 were digested with *SpeI* and *KpnI* (New England Biolabs). The digested products were ligated using T4 DNA ligase (Fermentas). The modified pFA545 including the AAC6′-APH2′ gene (pFA545gen) was transformed into *E. coli* DH10 competent cells (Invitrogen), amplified (selected on ampicillin 100 mg/l or gentamicin 4–8 mg/l) and purified using the QIAprep spin column (Qiagen). An *EcoRV* (Fermentas) digest was performed on the purified original pFA545 (predicted digest products 7729 bp, 2038 bp, 312 bp → giving a total size of 10,079 bp) and the modified pFA545gen (predicted digest products 10,432 bp, 312 bp → 10,744 bp in total) and band patterns were compared on a 0.8% agarose gel (data not shown). pFA545gen was transformed into *S. aureus* RN4220 by electroporation.

**Table 1:** Primers.

Name	Sequence (orientation 5′ - 3′)	Source
**Forward primer ** ***KpnI***	GTGGGTACCTTAAFCCTAGAGCTTGCCATGTATATG	This study
**Reverse primer ** ***SpeI***	CTCACTAGTGTCTGGACTTGACTCACTTCC	This study
**254 oligo**	CGACTGGACCTGGA	J. H. Wang
**256 oligo**	GATAAGCAGGGATCGGAACCTCCAGGTCCAGTCG	J. H. Wang
**ForwardTnL**	CTTAAGTTTGCTTCGATGACTGG	This study
**Reverse primer 258**	GATAAGCAGGGATCGGAACC	J. H. Wang
**ErmB forward 26**	GGAACATCTGTGGTATGGCG	This study
**ErmB reverse 27**	CATTTAACGACGAAACTGGC	This study
**Transposon-specific primer**	AATGATACGGCGACCACCGAGATCTACACCTGAATTACCCTGTTATCCCTATTTAGGTGAC	Langridge *et al.* (2009)
**P5**	AATGATACGGCGACCACCGA	Illumina
**P7**	CAAGCAGAAGACGGCATACGA	Illumina
**Sequencing primer**	GACACTATAGAAGAGACCGGGGACTTATCAGC	This study

The table lists the primers used in the experimental approach. It includes primer name, nucleotide sequence and orientation, and source.

### Construction of transposon mutant library

pMARGK2b and pFA545gen were transduced into *S. aureus* ST398 S0385 in two separate rounds of transduction using the *S. aureus* bacteriophage 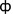
11. Donor cells (SH1000 pMARGH2b or RN4220 pFA545gen) grown to mid-exponential phase OD_600_ 0.5–0.8 were mixed in a 1∶1 ratio with two fold dilutions of phage in a 0.9% NaCl solution enriched with 10 mM CaCl_2_. Following 5 min absorption at room temperature (rt.), the cells were plated in a TSB-top-agar solution (TSB, 0.5 mM CaCl_2_, 0.5% agar) onto TSA plates supplemented with the appropriate antibiotics and incubated at 30°C over night. Top agar from plates with high phage titre were isolated, centrifuged (7,000 rpm, 10 min.) and sterile filtered using a 0.45 µm Millipore filter. Recipient cells (*S. aureus* ST398 S0385) were grown to OD_600_ 1–1.2, cells harvested by centrifugation (11,000 rpm, 10 min.) and re-suspended in TSB with 0.5 mM CaCl_2_. Prepared recipient cells and phage lysate were mixed in different ratios (100∶1–100∶15), incubated at rt. for 5 min, followed by the addition of 0.5 mM CaCl_2_ and incubated additionally 20 min. at rt. 0.02 M ice cold sodium citrate was added and mixed by vortexing. Cells were isolated by centrifugation (4000 rpm, 20 min, 4°C), re-suspended in 0.02 M sodium citrate, plated and incubated on BHI agar enriched with 0.2 mM sodium citrate and the appropriate antibiotic at 30°C over night. Transductants were sub-cultivated on selective plates containing the appropriate antibiotics and tested in an *ermB* and AAC6′-APH2′ PCR. Transductant, MRSA ST398 S0385 pMARGK2b pFA545gen was cultured at 30°C (plasmid replication at ≤30°C) with aeration in BHI supplemented with 5 mg/l erythromycin and 16 mg/l gentamicin and stored at −80°C in 0.5 ml aliquots (>10^6^ cells) with 50% glycerol.

The transposon mutant library was generated as described by Chaudhuri et al. (2009) with some modifications [8]. A 0.5 ml aliquot was inoculated into 100 ml BHI containing 5 mg/l erythromycin and chloramphenicol (Sigma) and 16 mg/l gentamicin and incubated at 30°C with aeration until the culture reaches OD_600_ 0.4. Cells were recovered from 30 ml culture by centrifugation (4000 rpm, 10 min) and re-suspended in 600 ml BHI containing 5 mg/l erythromycin pre-warmed to 43°C. The culture was grown at 43°C with aeration until the culture reached an OD_600_ 0.4. 30 ml culture was recovered by centrifugation (4000 rpm for 10 min) and re-suspended in 600 ml BHI containing 5 mg/l erythromycin pre-warmed to 43°C and the culture was grown at 43°C with aeration over night. The following day 30 ml culture was recovered and re-suspended in 600 ml BHI containing 5 mg/l erythromycin pre-warmed to 43°C and grown at 43°C with aeration over night and the same procedure was repeated one more day resulting in a 3^rd^ generation transposon mutant library. Each day cells were plated on BHI plates containing 5 mg/l erythromycin, 5 mg/l chloramphenicol or 16 mg/l gentamicin and grown at 37°C over night. The growth pattern demonstrated a 100% cure of pMARGK2b, ∼93% cure of pFA545gen and successful transposition of the transposon. Transposon mutants were stored in 0.5 ml (>10^6^ cells) 50% glycerol aliquots at −80°C until further use.

### Mutant library verification

Linker PCR was used to verify the complexity of the generated transposon mutant library. DNA was extracted (Gram positive DNA extraction Epicentre – lysing the cells with Ready-Lyse Lysozyme over night) from the transposon mutant pool in addition to DNA from 15 randomly isolated colonies (BHI plates containing 5 mg/l erythromycin) representing 15 random transposon mutants from the library. The DNA was digested with *RsaI* (Promega) and purified using a Minielute PCR purification kit (Qiagen). Adaptor molecules were made by mixing a 1∶1 ratio (100 µM) of oligo 254 and 256 (see Table 1), denatured at 95°C for 3 min. in annealing buffer (10× annealing buffer  = 100 mM Tris pH8, 500 mM NaCl, 10 mM EDTA) and annealed at room temperature for 1 hour (store at −20°C). Adaptors and digested DNA were ligated using a Quick DNA Ligase (New England Biolabs) followed by purification using a PCR purification kit (Qiagen). A PCR with primers ForwardTnL and reverse primer 258 (see Table 1) and Hotstar taq polymerase (Qiagen) was conducted with the following conditions: Hot-start 15 min at 95°C, 30 cycles of denaturation for 45 sec at 94°C, annealing 1 min at 55°C and elongation for 2 min at 72°C and a final elongation for 5 min at 72°C. The PCR products were visualised on a 2% NuSieve GTG Agarose gel (Lonza) (3 hours, 100 volts).

### Passage of transposon mutant library in broth

A 0.5 ml mutant library aliquot (>10^6^ cells) was inoculated in 10 ml BHI supplemented with 5 mg/l erythromycin and incubated over night at 37°C with aeration. 500 µl of the culture was re-inoculated into fresh BHI supplemented with 5 mg/l erythromycin and incubated over night at 37°C with aeration. The passage of the transposon mutant library was repeated three times. After each passage the library was tested for viable counts (results not shown) and DNA (from ∼10^9^ cells) was extracted using Easy-DNA kit (Invitrogen) which was stored at −20°C.

### Ethical statement

The study protocol was submitted to the ethical review committee at the University of Cambridge, Department of Veterinary Medicine, who reported that post mortem collection of blood following the slaughter of male pigs, surplus to a breeding program, is not a regulated procedure and provided ethical approval. The UK Animals (Scientific Procedures) Act 1986 allows for the use of animal tissues and blood in research that comes from animals not regulated by the Act. These animals were slaughtered by a method of killing identified in Schedule 1 of the Act. In this case, a 6-month-old male pig was euthanized by intravenous overdose of pentobarbitone and the blood was collected immediately postmortem into heparinised containers after obtaining the farm owner's permission for the use of their pigs in this study.

### Whole porcine blood survival

Two 50 ml falcon tubes were filled with approximately 10 ml heparinised whole porcine blood and each tube was inoculated with 0.5 ml mutant library aliquot (8.8×10^7^ cells). DNA was extracted from pooled mutant library aliquots (∼10^9^ cells) using MasterPure Gram Positive Purification Kit (Epicentre) and stored as input pools (replicates) at −20°C. The blood samples were incubated for 24 hours at 37°C with aeration. The following day the blood cultures were tested for viable counts (1.4×10^7^ CFU/ml) and 500 µl (∼10^7^ cells) from each blood-culture were inoculated into 2× 10 ml BHI supplemented with 5 mg/l erythromycin, to increase the bacterial/blood cell ratio prior to DNA extraction, and incubated over night at 37°C with aeration. This resulted in two rounds of growth selection, one selection round in whole porcine blood followed by a selection round in BHI. After the second round of selection DNA was extracted from ∼10^9^ of the mutants and stored at −20°C as output pools (replicates).

### Library preparation for Illumina sequencing

For the TraDIS approach the library preps were prepared as described by Langridge *et al.* (2009) with modifications [9]. 3–5 µg of DNA from input and output pools were fragmented to an average size of approximately 200 bp by Covaris E210. The size profile was evaluated with Agilent 2100 Bioanalyzer on a DNA1000 chip. The fragmented DNA was prepared for sequencing on an Illumina platform using the SureSelect XT Library Prep Kit-ILM (Agilent). The ligated fragments were amplified using a transposon-specific primer (see Table 1) and the multiplexing PCR primer index 1–8 supplied in the SureSelect Library Prep Kit. The PCR was run for 22 cycles with 200–400 ng template-DNA per reaction to amplify the transposon insert and junction sites. The PCR products were cleaned using 0.8× Agencourt AMPure XP beads (Ramcon) to remove DNA fragments below 200 bp. The quality of the amplified products was assessed using an Agilent 2100 bioanalyzer on a high Sensitivity DNA chip and quantified by Q-PCR with primers P5 and P7 (see Table 1). The libraries were pooled in a 1∶1 molar ratio and sequenced on an Illumina Hiseq2000 platform for 43 cycles plus index read using a custom sequencing primer (see Table 1) resulting in reads with the initial 10 bp being transposon insert specific followed by the junction region.

### Sequencing analysis and statistics

Sequence reads from the Illumina FASTQ files were sorted by index and evaluated for the 10 bp transposon (Tn) sequence CAACCTGTTA allowing 1 mismatch, using the program Sabre (https://github.com/najoshi/sabre). The Tn and adapter sequences, as well as short reads (<10 nucleotides) and nucleotides with poor base call quality (<Q15), were stripped using Cutadapt [22] and the junction regions were extracted and mapped to the reference genome (AM990992) using Bowtie 2.0 [23]. An in-house script was used to identify the precise transposon insertion sites and quantify the number of reads mapping to the open reading frames within the reference genome. The program Circos [24] was applied for a genome wide visualization of the transposon mutant library.

The number of unique transposon insertion sites for any given gene was calculated and divided by the average gene length using an in-house script (insertion index calculation). Genes with zero or few transposon insertions sites were categorised based on function using the COG (Cluster of Orthologous groups) database [27], [28], as described in Khatiwara *et al.* (2012) [10]. They were plotted as a percentage of all the COG categorised genes encoded by the reference genome.

The transposon mutant library was screened in whole porcine blood *in vitro* and mutants from input and output pools were compared using the DESeq package in R [25] enabling identification of significant differences in mutant composition pre- and post- selection. The approach was as described in Anders and Huber (2012) [26] and the settings are defined in Figure S1. The read counts, corresponding to transposon insertion sites were normalized to account for variation in the total number of reads obtained from each samples. The ratio of input:output read counts were determined and referred to as a log_2_ fold change. A negative log_2_ fold change reflects an attenuated mutant whereas a positive log_2_ fold change mirror a hypercompetitive mutant. For each individual mutant, the hypothesis that the fitness score was equal to zero and thereby that the mutant was present at equivalent levels in the input and output pools was tested for, using a negative binomial distribution as implemented in DESeq. The model was fitted only from those mutants from which replicate data was available and the resultant model was then applied to data derived from all mutants to estimate *P* values. An attenuated mutant was determined when the number of read counts from input pool to output pool significantly decreased and a hypercompetitive mutant was determined when the number of read counts from input to output pool significantly increased.

The raw sequence data will be available in the NCBI Sequence Read Archive (SRA) upon publication (Accession: SRR1056406 - SRR1056422).

## Results

### A construct for manipulation of LA-MRSA ST398

The transposon mutant library was generated in the whole genome sequenced wild type LA-MRSA ST398 S0385 isolate using a two plasmid system. One of the plasmids carried a Tn5 derived transposon with an erythromycin resistance marker and mariner mosaic ends, which was required for use in *S. aureus*. As most LA-MRSA ST398 harbour natural resistance to tetracycline, the tetracycline resistance marker in the transposase-bearing plasmid was substituted with a gentamicin resistance cassette, as S0385 was found, by susceptibility testing to be susceptible to gentamicin (Minimal Inhibitory Concentration, MIC = 0.5 mg/l). The tetracycline resistance gene was removed from the plasmid and the AAC6′-APH2′ gene originating from MRSA MU50 encoding gentamicin resistance was inserted into the plasmid at a position that facilitate the usage of the tetracycline resistance gene promoter. The plasmids were successfully transduced into the S0385 isolate.

### Transposon mutant library

A high complexity mariner transposon mutant library was generated in the whole genome sequenced wild type LA-MRSA ST398 S0385 isolate. Serial dilution and plating on BHI agar plates containing the appropriate antibiotic determined a mutant library size of ∼10^6^ mutants, a 100% plasmid loss of the transposon carrying plasmid and approximately 93% plasmid loss of the transposase-carrying plasmid. Due to the incomplete loss of the transposase bearing plasmid, nutrient-rich broth was supplemented with erythromycin at each growth step to ensure that the genomic insertion of the transposon was maintained. Linker PCR and DNA sequencing was used to verify transposon insert throughout the bacterial genome (Figures S2 and S3).

### Validation of the mutant library

DNA was isolated from the raw transposon mutant library and prepared for Illumina sequencing and sequenced on the HiSeq2000 platform. The sequencing was performed using a custom sequencing primer, sequencing from the 5′ end of the transposon and into the genomic DNA flanking the transposon insert.

In a sequencing run, one lane from an Illumina flow cell generated a minimum of 40 million reads of 43 bp plus index reads. The first 10 bp of each read constitutes the Tn sequence. Each lane was multiplexed with seven or eight samples, resulting in a minimum of 165 million nucleotides that represent the actual target DNA per sample. *S. aureus* S0385 has a total of 2777 annotated genes with an average length of 874 bp resulting in an average of 67x gene-coverage.

One mismatch was allowed when matching the Tn sequence. When using the HiSeq platform a lower quality of the Tn sequence was obtained in comparison to the quality of the target regions, as the Tn sequence is identical in all the reads. The sample used for validation had a total output of ∼7.1 million reads and of these the Tn sequence was identified in ∼6 million reads. Tn sequence and adapter sequence were stripped and the reads (10–23 bp in length) were mapped to the reference genome. ∼4.5 million reads were mapped exactly one time and 140,330 unique insertion sites were identified. The average distance between unique insertion sites was 20.5 bp and by utilising an average gene length of 911 bp (average gene length for genes containing an insert), the average number of unique inserts per gene was 44.8. The top row of Table 2 shows an overview of the transposon inserts recovered from the raw mutant library.

**Table 2:** Overview of the raw Transposon mutant Library and the passages in BHI - Illumina sequence data.

	Total no. of reads	Read with Tn tag (≤1 mismatch)	Reads mapped exactly 1 time	No. of unique insertion sites	Average no. of unique insertion sites per gene
**Raw library**	7,129,995	6,070,601	4,503,675 (75.88%)	140,330	44.8
**Passage 0**	7,564,547	5,931,390	4,284,574 (73.97%)	136,440	42.4
**Passage 1**	10,503,621	8,586,527	6,003,415 (71.14%)	97,236	31.2
**Passage 2**	10,316,723	8,481,909	6,017,839 (72.35%)	115,921	37
**Passage 3**	13,618,447	11,261,919	7,899,885 (71.54%)	162,228	51

The table shows the output from the raw transposon mutant library and the three passages in BHI. The number of reads recovered after trimming and alignment were identified and the number of unique insertion sites per gene was calculated. The sequence data of the raw mutant library was obtained from one lane of a flow cell which was multiplexed with eight samples. The sequence data from the three passages were obtained from one lane of a flow cell that was multiplexed with seven samples. The sequencing was performed on a Hiseq2000 platform.

**Figure 1:**
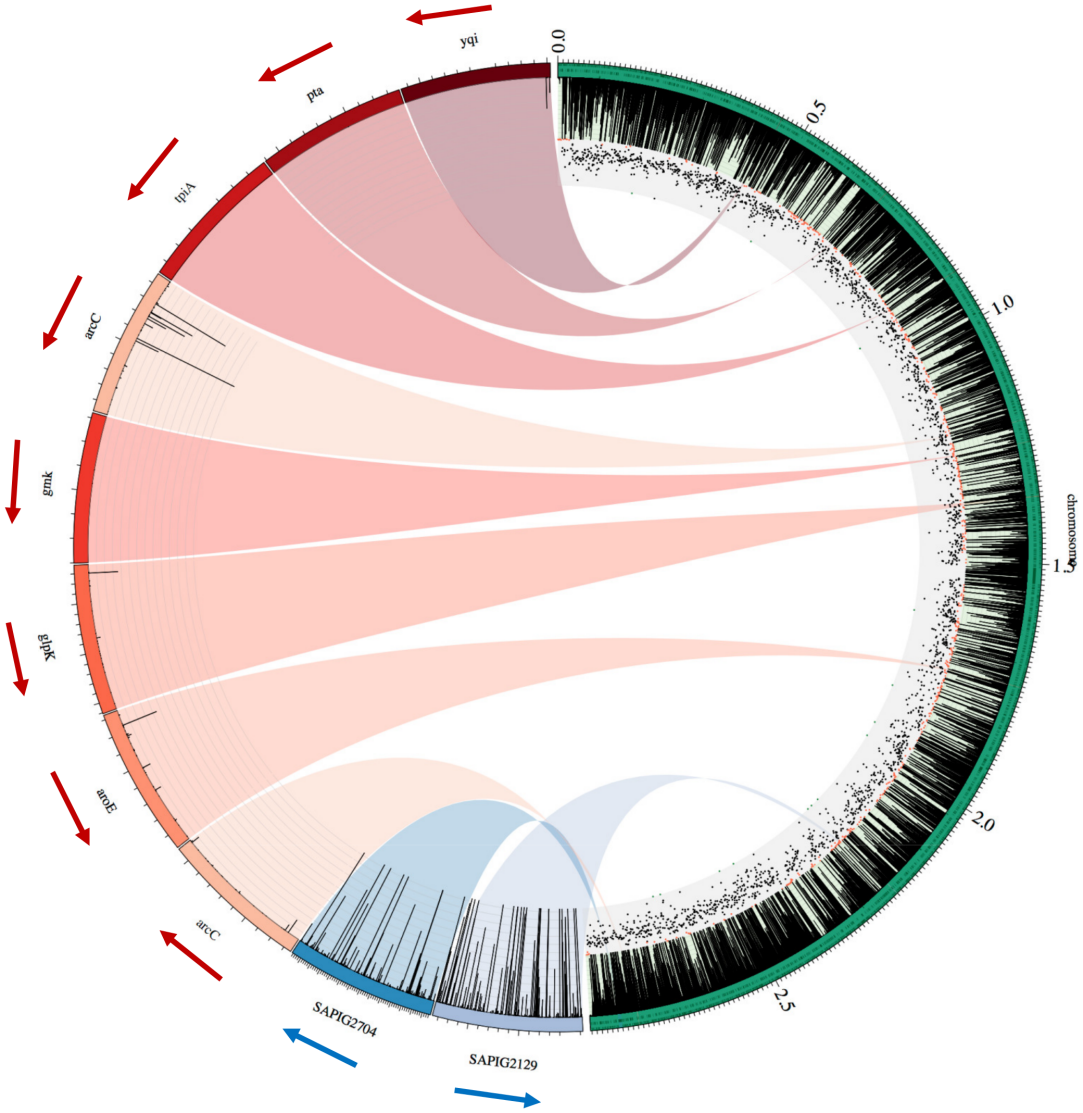
Genome atlas. Right semicircle: The green band in the outermost part of the semicircle illustrates the reference chromosome (AM990992) with the size of 2,872,582 bp. The three circular plasmids harboured by the reference are not included. The black spikes connected to the green semicircle shows the distribution of the reads from the raw transposon mutant library aligned to the reference strain. The black and red dots indicate positions within the reference with large number of reads (insertion index >0.02) and low number of reads (insertion index <0.02) respectively. Left semicircle: The red colours show of zoom of the seven MLST genes (*arcC* represented twice due to two copies of this particular gene) and the black spikes illustrated in some of the genes show reads mapping within the open reading frame. The arrows indicate transcription direction. The zoom of SAPIG2704 and SAPIG2129, visualised in blue colours, show examples of two genes with a large number of read mapping throughout the open reading frames.

The distribution of the reads aligned to the reference chromosome is illustrated in Figure 1 by the right semicircle of the genome atlas. Reads are demonstrated as black spikes that are aligned to the reference genome, which is illustrated by the outermost green circle. The distribution of the aligned reads shows a high complexity transposon mutant library with inserts throughout the chromosome and no specific hotspots for transposon insertion.

**Figure 2:**
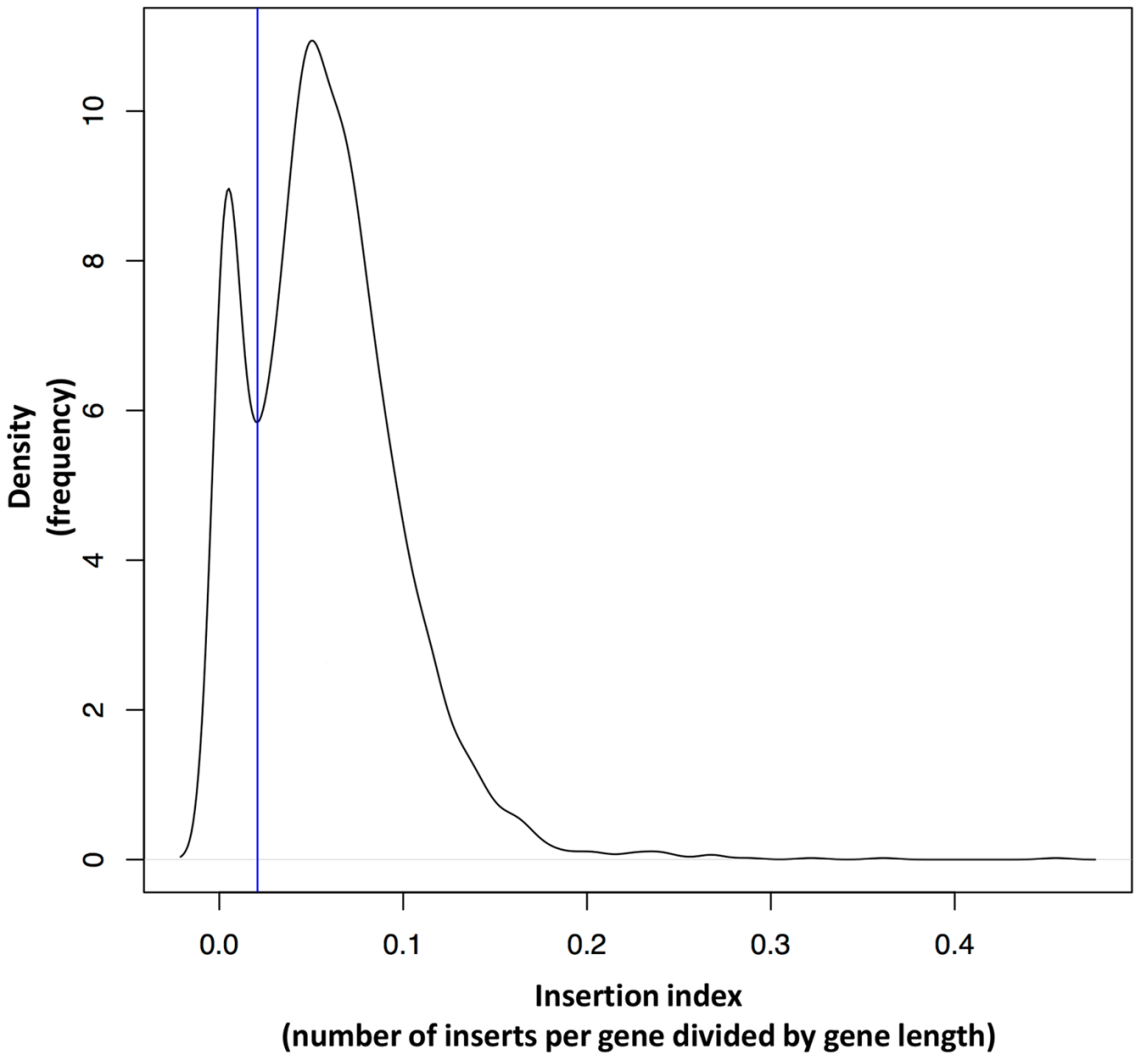
Density plot - Insertion index distribution. The figure shows a density plot illustrating the distribution of insertion indices (number of transposon inserts per gene divided by an average gene length). The plot indicates the density according to which the insertion indices are distributed and it shows that the insertion indices have a bimodal frequency distribution. The leftmost peak represents the genes with zero or very few insertions, whereas the rightmost peak represents the genes with a large number of insertions. The vertical line piercing the local minimum and separating the two peaks, defines the cut-off sorting genes as either, essential/beneficial or non- essential/neutral for bacterial fitness in a given environment.

Transposon insertion into a non-functional part of a gene may not disrupt gene function so it is necessary to define a threshold to separate essential/beneficial genes from non-essential genes. An insertion index was calculated by dividing the number of unique insertion sites for any given gene by the average gene length for genes containing an insert. Figure 2 illustrates a density plot based on the calculated insertion index for each gene. This plot separates genes with a low number of transposon inserts from genes with a high number of inserts (see Figure 2). The left most peak shows genes with a low number of inserts representing mutants with a decrease in fitness, which could lead to total loss of cellular survival or an arrested cell cycle, whereas the right most peak illustrates genes with a high number of inserts, representing viable mutants. The local minimum separating the peaks suggests that a cut-off value of around 0.02 would be suitable to distinguish essential/beneficial genes from non-essential genes.

The seven housekeeping genes *aroE*, *glpK*, *gmk*, *pta*, *tpiA*, *yqiL* and *arcC* used for Multi Locus Sequence Typing (MLST), shown in red in the left semicircle of Figure 1, represent potential candidates of essential genes within the *S. aureus* genome. One of the MLST genes (*tpiA* (SAPIG0853)) mapped zero reads, four genes mapped few reads (*pta* (SAPIG0662), *gmk* (SAPIG1207), *yqiL* (SAPIG0434) and *glpK* (SAPIG1302)) resulting in insertion indices below the cut-off (<0.02), identifying five of the MLST genes as essential/beneficial using this system. *aroE* (SAPIG1661) and *arcC* had insertion indices above the cut-off defining them as non-essential.

SAPIG2704 and SAPIG2129 (see Figure 1), shown in the left semicircle of Figure 1, encode serine-rich adhesin for platelets and cardiolipin synthetase, respectively, and are examples of two non-essential genes from the S0385 genome. A high number of reads mapped to these open reading frames, indicating that there was no significant loss of fitness when these genes were disrupted by transposon insertions.

### LA-MRSA ST398 genes important for growth

The mutant library was grown for three passages in nutrient-rich broth at 37°C to identify genes essential for growth in this substrate. Table 2 shows an overview of the sequence analysis from passage 0 to passage 3. 71–75% of the reads containing the Tn tag sequence were found to map the reference genome. The number of unique insertion sites was between 97,000 and 162,000 with 31–51 unique insertion sites per gene. The number of unique insertion sites showed an initial decrease between passage 0 and passage 1. The decrease could illustrate that the transposon mutant library contains slow growing mutants, which will not be identified in the first growth passage. The passages were performed 3 times to increase selection sensitivity and to reduce the presence of arrested and dead cells.

**Figure 3:**
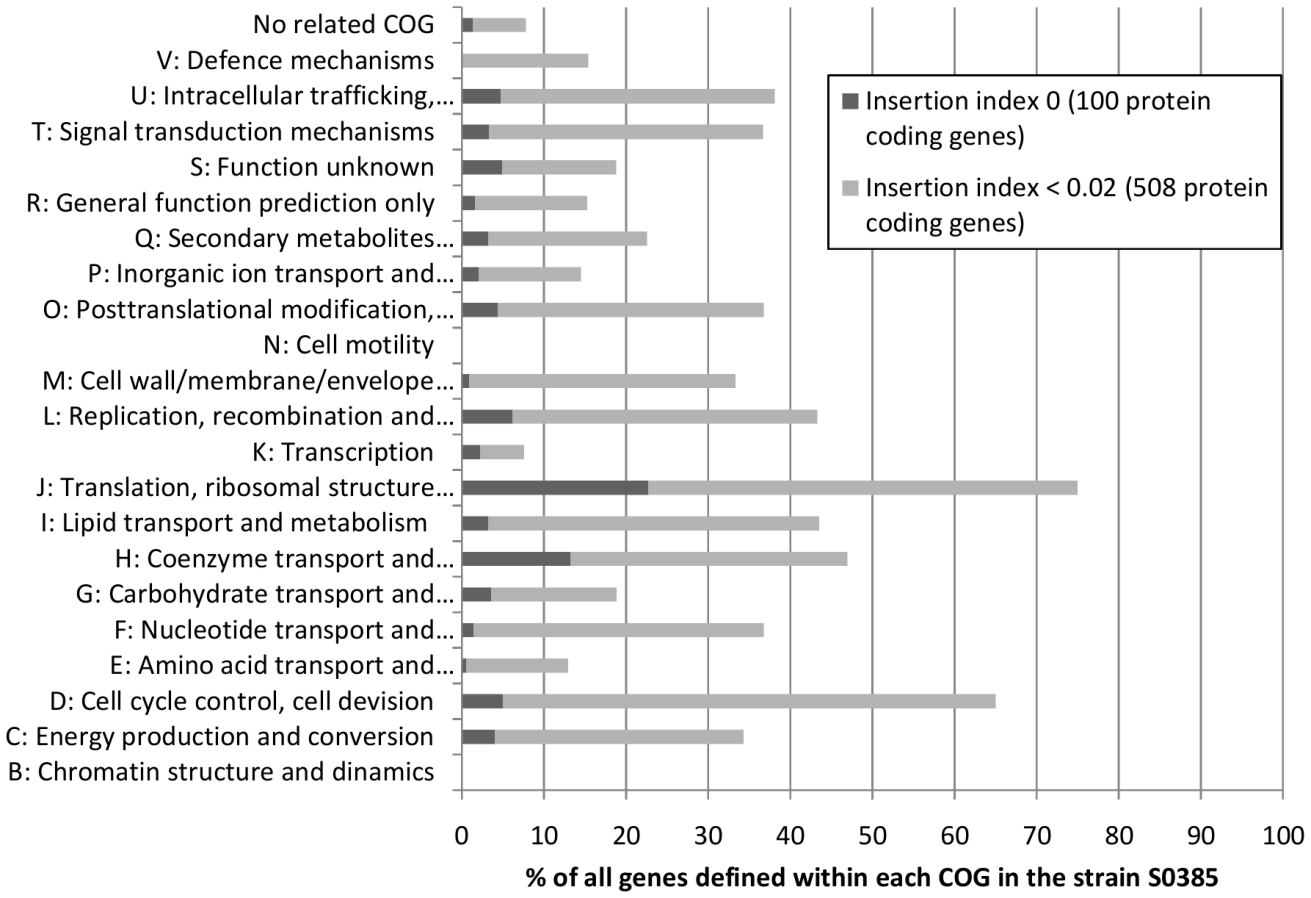
Proposed essential genes classified by functionality. The proposed essential genes for growth under laboratory conditions were classified by functionality and plotted as a percentage of all genes within each functional group encoded by the reference strain. The genes were assigned a functionality based on the COG database and these groups are illustrated on the vertical axis. The dark grey columns represent the proposed essential protein-coding genes with zero inserts, whereas the light grey columns add the protein-coding genes with few inserts (insertion index <0.02), which were proposed beneficial for growth under laboratory conditions.

A total of 152 genes mapping zero reads were identified from the mutant pool after three passages under laboratory conditions – of these, 100 were protein-coding genes, 4 encoded ribosomal RNAs (rRNA) and 48 transfer-RNAs (tRNAs). These genes are proposed to be essential for bacterial survival under laboratory conditions. In addition, 526 genes had only a few mapped reads and had an insertion index below the calculated cut-off of 0.02, indicating that these may also be important for growth (Tables S1 and S2). Genes with few transposon insertion sites may have maintained gene but cannot be identified as true essential genes and are therefore referred to as genes beneficial for bacterial survival under laboratory conditions. The protein coding genes were categorised based on functionality using the COG database and plotted as percentage of all the COG categorised genes in the WT (see Figure 3). Some genes were categorised as belonging to several COGs. The proposed essential gene list includes representatives of all the major functional COGs except group B (chromatin structure and dynamics) and N (cell motility). Representatives in V (defence mechanisms) were only identified when including the genes with few inserts (insertion index <0.02). Protein-coding genes involved in translation (COG group J), cell division (COG group D), coenzyme transport and metabolism (COG group H), and intracellular trafficking, secretion and vascular transport (COG group U) had the largest number of representatives in the proposed essential and beneficial gene sets. Approximately 9% of the proposed essential and beneficial protein-coding genes were of unknown function or not related to any COG group.

### Survival in whole porcine blood

The mutant library was grown in porcine blood and DNA from mutants pre- and post- selection was prepared as input and output pools, respectively. The blood samples were inoculated with the transposon mutant library and incubated for 24 hours. Previous growth experiments in whole porcine blood showed an initial decrease in cell counts but after 24 hours of incubation the number of mutants returned to a population size equivalent to the inoculums (see Figure S4 for more details). The total number of reads corresponding to transposon insertion sites in the input pool was compared to the total number of reads mapping to the equivalent position in the output data. The read counts are expected to follow an approximately normal distribution but the data showed some noise in the lower end and read counts below 2^4^ were considered as noise based on a frequency distribution plot (data not shown). Using the DESeq package in R the effective size of each sequence library was estimated based on the read counts and the estimated size factors were used for normalization of the data. To contrast the two conditions and highlight a possible differential composition in mutants, recovered pre- and post- selection, the variance of reads mapping each gene was estimated and subsequently tested using a negative binomial test. Ratios of normalized read counts in the input and output samples were determined and expressed as a log_2_ fold change. A negative log_2_ fold change corresponds to a decrease in read counts from input to output and indicates attenuated mutants, whereas a positive log_2_ fold change reflects an increase in read counts from input to output.

Only the mutants that were uniquely attenuated under the selective conditions were of interest. The mutant composition pre-selection in whole porcine blood was compared to the mutant composition post-selection. The genes representing the mutants with the most significant change in clone number were identified. To eliminate general selection due to growth in BHI the mutant library was selected for an equivalent number of growth rounds in BHI and genes representing mutants with the most significant change in clone number were identified. The two gene lists were compared and the genes specific for survival in whole porcine blood were identified (see Figure S5).

Transposon inserts in 23 genes induced a significant decrease in fitness (negative log_2_ fold change) and transposon inserts in one gene induced a significant hypercompetitive mutant (positive log_2_ fold change), all as a consequence of being selected in porcine blood (see Table 3). Six of the mutants, illustrated with a minus infinity (-inf) log_2_ fold change in Table 3, were represented in the input pool but totally absent, with zero read counts, in the output pools. Seven of the 23 genes are defined as encoding hypothetical proteins with unknown function. Additionally two genes were of unknown function, whereas fifteen could be assigned a potential function (see Table 3 and Table 4).

**Table 3:** Genes representing 23 attenuated mutants and 1 hypercompetitive mutant when selected in whole porcine blood.

ID (gene)	Read Count Input	Read Count Output		
Mean	Mean	Log2	Fold Change	*P*-value
**SAPIG2099**	428.16	0.00	-inf	0.0237
**SAPIG1465**	317.79	0.00	-inf	0.0156
**SAPIG2108**	203.34	0.00	-inf	0.0288
**SAPIG0429**	196.84	0.00	-inf	0.0298
**SAPIG1848**	164.21	0.00	-inf	0.0354
**SAPIG0633**	143.38	0.00	-inf	0.0398
**SAPIG0142**	726.16	1.10	−9.3631	0.0024
**SAPIG1650**	492.68	1.10	−8.8088	0.0128
**SAPIG1041**	799.62	2.20	−8.5048	0.0025
**SAPIG1748**	352.17	1.10	−8.3244	0.0249
**SAPIG1921**	306.01	1.10	−8.1163	0.0430
**SAPIG0315**	605.24	2.21	−8.1003	0.0090
**SAPIG2670**	256.41	1.10	−7.8613	0.0406
**SAPIG2057**	469.89	2.21	−7.7351	0.0203
**SAPIG1726**	853.28	4.40	−7.5998	0.0038
**SAPIG1977**	526.66	3.30	−7.3165	0.0272
**SAPIG0258**	446.43	3.31	−7.0762	0.0426
**SAPIG1054**	1430.32	12.11	−6.8836	0.0019
**SAPIG1096**	694.20	6.60	−6.7168	0.0137
**SAPIG2156**	529.71	5.51	−6.5882	0.0296
**SAPIG0647**	1739.26	31.92	−5.7678	0.0497
**SAPIG2568**	751.50	15.43	−5.6056	0.0259
**SAPIG2639**	1449.27	77.16	−4.2314	0.0360
**SAPIG0185**	120.11	2868.20	4.5777	0.0328

The table lists the mutants that significantly changed in clone number from input to output (pre- and post-selection in whole porcine blood). The top 23 genes represent the mutants that were significantly reduced in number of clones after selection in whole porcine blood. The lowermost gene represents the mutant that significantly increased in clone number after selection in whole porcine blood. Mean read count input and Mean read count output represent the mean number of reads mapping within the defined gene. The differences between the mean values are illustrated by a log fold change from input to output and a negative log_2_ fold change indicating changes in fitness. A negative log_2_ fold change defines attenuation in fitness whereas a positive log_2_ fold change defines increase in fitness. The p-value shows the level of significance.

**Table 4:** Description of the genes identified as important for *S. aureus* ST398 survival in whole porcine blood.

ID (gene)	Description	Process	Whole blood survival
**SAPIG2099 (** ***leuD*** **)**	3-isopropylmalate dehydratase, small subunit	Leucine biosynthesis (amino acid biosynthesis)	Oxidative stress and pH shock. Stringent response (cellular adaptation to nutrient limiting conditions).
**SAPIG1465 ** ***(aroB)***	3-dehydroquinate synthase	Nucleotide and amino acid metabolism (aromatic amino acid metabolism)	Oxidative stress and pH shock.
**SAPIG0429**	Hypothetical protein	Unknown	?
**SAPIG2108**	Phosphoserine phosphatase, RsbU	Up-regulation of σ^B^ (alternative sigma factor)	σ^B^ influences expression of a variety of genes including virulence genes under stress and specific environmental conditions.
**SAPIG1848**	Hypothetical protein	Unknown	?
**SAPIG0633**	tRNA-specific adenosine deaminase	Unknown	?
**SAPIG0142**	NAD dependent epimerase/dehydratase family protein	Galactose metabolism	Glucose depletion. Galactose metabolism (galactose molecules compose important components of the surface bound antigens located on red blood cells).
**SAPIG1650 ** ***(lepA)***	GTP-binding protein	Specific function unknown	LepA protein homologous to translation factors that binds ribosomes.
**SAPIG1041 ** ***(menD)***	2-succinyl-6-hydroxy-2, 4-cyclohexadiene-1-carboxylic acid synthase/2-oxoglutarate decarboxylase	Menaquinone biosynthetic pathway	Respiration. Involved in protection against haem toxicity
**SAPIG1748 ** ***(icd)***	Isocitrate dehydrogenase (IDH), NADP-dependent (icd gene)	Regulation of tricarboxylic acid (TCA) cycle	Icd up-regulation under acidic conditions. Regulation of the TCA cycle.
**SAPIG1921**	RNA methyltransferase, TrmH family, group 2	RNA metabolism	Regulation – balance between transcript and degradation of mRNA.
**SAPIG0315**	Hypothetical protein	Unknown	?
**SAPIG2670**	Hypothetical protein	Unknown	?
**SAPIG2057**	Aspartate transaminase	Aminoacid metabolism.	Decrease in pH.
**SAPIG1726**	HemA concentration negative effector hemX	Transport	ABC-type transport system. C ytochrome c biogenesis.
**SAPIG1977**	Response regulator protein VraR	Regulator of cell wall damage stress response	Response to cell wall damage.
**SAPIG0258**	PTS system galactitol-specific enzyme II B component	Galactose metabolism	Glucose depletion. Galactose metabolism (galactose molecules compose important components of the surface bound antigens located on red blood cells).
**SAPIG1054**	Beta-lactamase		
**SAPIG1096**	Spermidine/putrescine ABC transporter ATP-binding subunit	ABC transporter involved in ion homeostasis	pH shock/changes.
**SAPIG2156**	Hypothetical protein	Unknown	?
**SAPIG0647**	Indigoidine systhesis protein	Secondary metabolite composing a blue pigment.	Oxidative stress – ph shock.
**SAPIG2568 ** ***(fbp)***	Fructose-1,6-bisphosphatase	Gluconeogenesis	Response to depletion of glucose.
**SAPIG2639 ** ***(pyrD)***	Dihydroorotate oxidase	Pyrimidine biosynthesis	Nucleic acids biosynthesis.
**SAPIG0185**	pANL51	Unknown function	?

## Discussion

The purpose of the work was to generate a high complexity transposon mutant library and assess the application of TraDIS in *S. aureus* ST398. LA-MRSA ST398 was selected for this study as it shows different host infection/colonization patterns compared to most other MRSA strains. The isolation of MRSA from animals was first reported in 1972 [29], but was at that time most likely associated with human to animal transmission of an MRSA strain acquired by the farmer during hospitalisation. More recently, a specific lineage belonging to CC398, most likely of human origin, has spread among livestock globally, acquired methicillin resistance and is now transferring back to humans leading to both colonisation and disease [17], [30]. ST398 is able to adapt to various host environments and continues to emerge worldwide both in livestock and also to some extent in hospital settings [31].

When interpreting the data it is important to recognize that the environment and other factors resulting from the experimental design can have unintended consequences on the output data. Nutrient-broth was supplemented with erythromycin to maintain the genomic insertion of the transposon and high temperatures were applied to promote plasmid loss, both of which may influence the output when screening for essential genes. For example it has been reported that incubation at high temperatures in the presence of erythromycin enriches for mutants of the *sae* system, which is a two-component system involved in regulation of some virulence genes [32]. Enrichment of a regulatory system could have an unattended effect on the transposon mutant composition. In addition, transposon insertions may affect the expression of downstream genes or operons, causing polar mutation that leads to incorrect identification of essential genes in a defined environment. For definitive identification of gene function it is necessary to generate single knockout mutants and test those in the same functional assays used in the screenings. However, since a large number of genes are listed as having no known function and there is inconsiderable value in generating evidence for the phenotypes resulting from the possession of these genes, high-throughput methods can help to narrow the pool of genes to be investigated further.

In our study we generated a transposon mutant library consisting of ∼10^6^ mutants and we identified around 140,000 unique insertion sites. The transposon mutant library generated in *Salmonella* Typhi by Langridge *et al*. (2009) [9] yielded 370,000 unique insertion sites, which may be explained by the fact that the *Salmonella* genome is more than 2 Mb larger than the *S. aureus* genome and so provides the potential for a higher number of unique insertion sites. Langridge *et al.* showed an average of one insertion site for every 15–20 bp, which was similar in this study showing an average insertion site for every 20.5 bp. The sequence data (Table 2), linker PCR data (Figures S2 and S3) and the coverage atlas (Figure 1) showed a successful generation of a high complexity mutant library with transposon inserts throughout the bacterial genome, comparable to the mutant library generation in *Salmonella* Typhi [9].

The MLST genes are housekeeping genes and are expected to be essential for cell viability [33]. However not all seven MLST genes were defined as essential in this study. The *glpK*, *gmk*, *pta*, *tpiA* and *yqiL* MLST genes were identified as essential or beneficial with zero or few transposon inserts, whereas *aroE* and *arcC* were defined as non-essential. *tpiA*, *pta*, *gmk* and *yqiL* have all been identified as essential previously (see Table S3) [6], [8], [12]. The *arcC* gene encoding carbamate kinase has a paralogous gene at a different locus within the S0385 genome, which also encodes carbamate kinase. When one of the *arcC* homologues is disrupted by the transposon insert the transcript of the other may take over and this could explain an insertion index above the cut-off for both *arcC* genes (SAPIG1164 and SAPIG2682).

Gene SAPIG2704 and SAPIG2129, which encode serine-rich adhesin for platelets and cardiolipin synthetase respectively, constitute two examples of genes defined as non-essential for S0385 survival under laboratory conditions in this study. Figure 1 illustrates that a high number of reads mapped within these open reading frames. Serine-rich adhesins are postulated to be important for bacterial binding to platelets as part of the pathogenesis in infective endocarditis in humans [34]. The S0385 isolate was isolated from a human case of endocarditis [35], where serine-rich adhesins may be essential, but when transferring the isolate to a laboratory environment, these adhesins might lose their importance for bacterial survival. Cardiolipin synthetase are involved in conversion of bacterial membrane phosphatidylglycerol (PG) to cardiolipin (CL) when the bacteria progress from exponential growth phase to stationary and when phagocytosed by human neutrophils [36]. The *S. aureus* S0385 genome contains two open reading frames (Cls1: SAPIG1324 and Cls2: SAPIG2129) encoding cardiolipin synthetases. Cls2 is primarily responsible for CL accumulation under stationary phase [36], but when SAPIG2129 encoding Cls2 is disrupted by transposon insert, the homologous Cls1 may take over. The examples above illustrate the sensitivity of this methodology for identifying essential/beneficial or non-essential genes.

A total of 152 *S. aureus* S0385 genes had zero transposon inserts and were therefore proposed as essential genes, while 526 genes, with a low number of transposon inserts, were proposed as beneficial for growth under laboratory conditions. Table S1 shows the lists of proposed essential genes and Table S2 the list of proposed beneficial genes. Table S3 shows a comparison with previously described *S. aureus* essential genes using high complexity transposon mutant libraries [6], [8], [12].

Of the 526 genes (insertion index <0.02) proposed here as beneficial, 268 genes have been described as essential in *S. aureus* previously (see Table S2). The 258 proposed beneficial genes that have not been described as essential previously encode proteins involved in DNA repair, replication and recombination, which indicate that the high temperatures applied to promote plasmid loss under the mutant library construction induced as expected bacterial stress conditions. These genes are therefore evaluated as beneficial for ST398 survival in this study due to the specific conditions applied in the experimental setup. When ranking the genes with insertion indices <0.02, it is clear that, as the insertion index increases and approaches the cut-off (0.02), there is an increase in number of genes that have not been described as essential in *S. aureus* previously (see Table S2). The ranking and knowledge from previous studies could indicate an insertion index cut-off of approximate 0.007 instead of 0.02. This shows that the selection of the cut-off separating essential/beneficial from non-essential genes is an important consideration.

The differences found between this study and previous studies defining essential genes could be due to differences in methodology, sensitivity of the methods, environmental conditions or true differences between bacterial strains. However, the results need to be verified by additional studies to provide further evidence of the essential nature of these genes.

None of the proposed essential genes were defined within the group of Defence mechanisms (COG group V), but four of the proposed beneficial genes were categorised as belonging to COG group V. These four genes, SAPIG1054, SAPIG1375, SAPIG1376 and SAPIG2314, encode beta-lactamase *ampC* and aminoacyltransferase *femA*, *femB* and *femX* respectively. *femA*, *femB* and *femX* have been identified as essential genes in previous studies [6], [8] and it has been shown that *femA* and *femB* mutants have a reduced peptidoglycan (PG) glycin content compared with *femA* ^+^ and *femB* ^+^ strains [37], [38]. The staphylococcal cell wall plays an important role in infection and pathogenicity, but based on our data these cell-wall impairments may also have wider influence on cell growth and survival in general. However, it has also been demonstrated that *femAB* null mutants harbouring an erythromycin resistance marker lead to a low level of erythromycin resistance, which may be due to a higher uncontrolled influx of erythromycin through the impaired cell-wall [39]. The presence of erythromycin in the nutrient-broth used in this study could explain the decreased fitness identified for the *femA*, *femB* and *femX* mutants.

Overall 24 genes were identified with a significant change in fitness after whole porcine blood incubation. Twenty-three of these genes were identified as giving a significant reduction in bacterial fitness when inserted with a transposon and selected *in vitro* in porcine blood. Mutation in one gene resulted in a hypercompetitive mutant post-selection in whole porcine blood.

No specific cell viability tests were performed on the blood cells, but it has been shown previously that whole-blood units stored at room temperature maintained cellular counts and coagulation activity for up to 72 hours [40]. In addition, in previous experiments an initial decrease in bacterial cell counts was observed when incubating the transposon mutant library in whole porcine blood, which could reflect neutrophil killing. It is therefore reasonable in this case to believe that the genes identified are important for survival in whole porcine blood under *in vitro* conditions.

The 23 genes identified in the attenuated mutants represent mutants showing the greatest reduction in cell count when comparing input and output pools. However, they are unlikely to be the only genes important for survival in porcine blood. For example, mutants with transposon inserts in essential genes are absent in the input pools and a potential difference between input and output pools for those essential genes will not be detected and they can therefore not be considered as important for whole porcine blood survival in this experiment.

Seven of the 24 genes are defined as hypothetical genes of unknown function and two other proteins were annotated with unknown function. Fifteen genes were annotated to be predominantly involved in carbon metabolism, pH shock, regulation and transport (see Table 4) [41]–[47]. This indicates that key genes for survival in porcine blood cultures may not be genes involved in iron uptake such as hemolysins and sideophors, but may be genes associated with the ability to utilize the available carbon hydrates in blood, regulation at different levels as well as survival under extreme pH conditions. This is supported by previous studies analysing global gene expression of *S. aureus* under *in vitro* conditions of short-term culture in human blood [48], [49]. In these studies, it was observed that up- or down regulated genes were mainly involved in cellular metabolism or had an unknown function. A previous study screening 1248 transposon *S. aureus* mutants in an *in vivo* murine bacteraemia model identified 50 genes as being important for whole blood survival, half of which had unknown function and the rest with an involvement in nutrient biosynthesis and surface metabolism [50]. Furthermore they identified genes important for the tricarboxylic acid cycle (TCA cycle) and in this study we identified the *icd* gene, a TCA cycle regulator, as important for *in vitro* survival in porcine blood. This indicates that the TCA cycle and carbon metabolism, have important functions for bacterial survival in blood *in vivo* and *in vitro* and in blood from different hosts. The *fem*A and *fem*B genes were previously identified as important for whole blood survival *in vivo* [50]. However, we found *fem*A and *fem*B mutants to have a growth disadvantage under laboratory conditions which is in correlation with other studies identifying *S. aureus* essential genes [6], [12], [13].

The transposon mutant library was incubated in whole porcine blood *in vitro* for 24 hours. This could partly reflect why many metabolic genes were identified as important for whole porcine blood survival in this study. However, an incubation period of 24 hours was specifically selected based on initial growth experiments in whole porcine blood *in vitro* (Figure S4). These experiments showed an initial decrease in bacterial population size, which could be explained by phagocytosis and potential bacterial killing by host immune cells. The mutant population size returned to an equivalent size of the inoculated population after 24 hours, and at this point the mutants had potentially seen all the selective elements within whole blood. Genes important for immune evasion will have undergone selection in a similar manner as the metabolic genes. *S. aureus* encodes however various immune evasion genes and it is justifiable to conclude that none of these are singlehandedly responsible for survival of the immune response, which could explain why none of these genes were identified as important for whole blood survival. Even though no specific virulence genes were identified as being important for blood survival in this study they might have important functions in more specific infection models.

In this study, we successfully generated a high complexity transposon mutant library in an LA-MRSA ST398 WT isolate and evaluated it using the TraDIS system. We identified *S. aureus* ST398 essential genes comparable with previous studies. Twenty-four genes were evaluated as being important for specific *in vitro* whole porcine blood survival, of which carbon metabolism, pH shock and regulation were related. For further evaluation of these genes, we aim to generate single knockout mutants and test these for survival in porcine blood, as well as in blood from other relevant donors. In addition, the generated transposon mutant library will be used in a screen for survival and colonization in other host relevant environments such as on porcine skin and nasal epithelium.

## Supporting Information

Figure S1 **Commands and settings used in R for the statistical analysis.**
(TIF)

Figure S2 **Whole mutant library and single colony verification.** The gels show the result of the linker PCR used for library validation. The left gel shows squared in red a low complexity mutant library with a laddering of the smears. The blue squared lanes illustrate the same high complexity transposon mutant library from passage 0 (lane 2) to passage 3 (lane 5). The third generation transposon mutant library shows a smear with no specific bands. The right gel represents 15 randomly picked single mutant colonies isolated from the third generation transposon mutant library, each giving a band of different size indicating that the transposon has inserted at different locations with the genome.
(TIF)

Figure S3 **Genome atlas identifying transposon inserts of 11 random isolated mutants.** The genome atlas illustrates by black marks in the outer most circle 11 different transposon insertion sites within the reference genome. The insertion sites were identified based on sequencing 11 of the 15 randomly picked mutant colonies described in figure S1. The fragments from the 11 mutants were sequenced and aligning to the reference genome. The blue and red parts of the atlas indicate forward and reverse transcriptional direction of the open reading frames within the reference genome.
(TIF)

Figure S4 **Growth profile of transposon mutant library in whole porcine blood** ***in vitro*** **.** The figure shows the growth profile of the transposon mutant population in whole porcine blood *in vitro*. Mutant population size was determined at specific time-points to identify functionality of the blood immune cells. After 24 hours incubation *in vitro* the mutant population size was equivalent to the inoculated population size (indicated by the red circle).
(TIF)

Figure S5 **Experimental setup for identification of genes important for bacterial growth in whole porcine blood.** The mutant composition in input pool pre-selection in whole porcine blood (Input pool - library aliquot) were compared with mutant composition in output pool post-selection in porcine blood (output pool BHI – second generation library). The mutants identified with a significant change in number of clones represent genes important for whole porcine blood survival in addition to growth BHI. The mutant composition in output pool post-selection in porcine blood (output pool BHI – second generation library) was compared to mutant composition after growth in BHI (BHI – second generation library). The mutants identified with a significant change in number of clones in both of the comparisons were evaluated as specific for survival in whole porcine blood *in vitro*.
(TIF)

Table S1 **Proposed essential genes.**
(XLSX)

Table S2 **Proposed beneficial genes.**
(XLSX)

Table S3 **Comparison of essential gene lists of** ***S. aureus*** **.**
(XLSX)
