# Supplementary figures and images for: Genome-Wide High-Throughput Screening to Investigate Essential Genes Involved in Methicillin-Resistant Staphylococcus aureus Sequence Type 398 Survival
Source: PLoS One. 2014 Feb 12;9(2):e89018. doi: 10.1371/journal.pone.0089018 (PMC3923074; doi:10.1371/journal.pone.0089018)

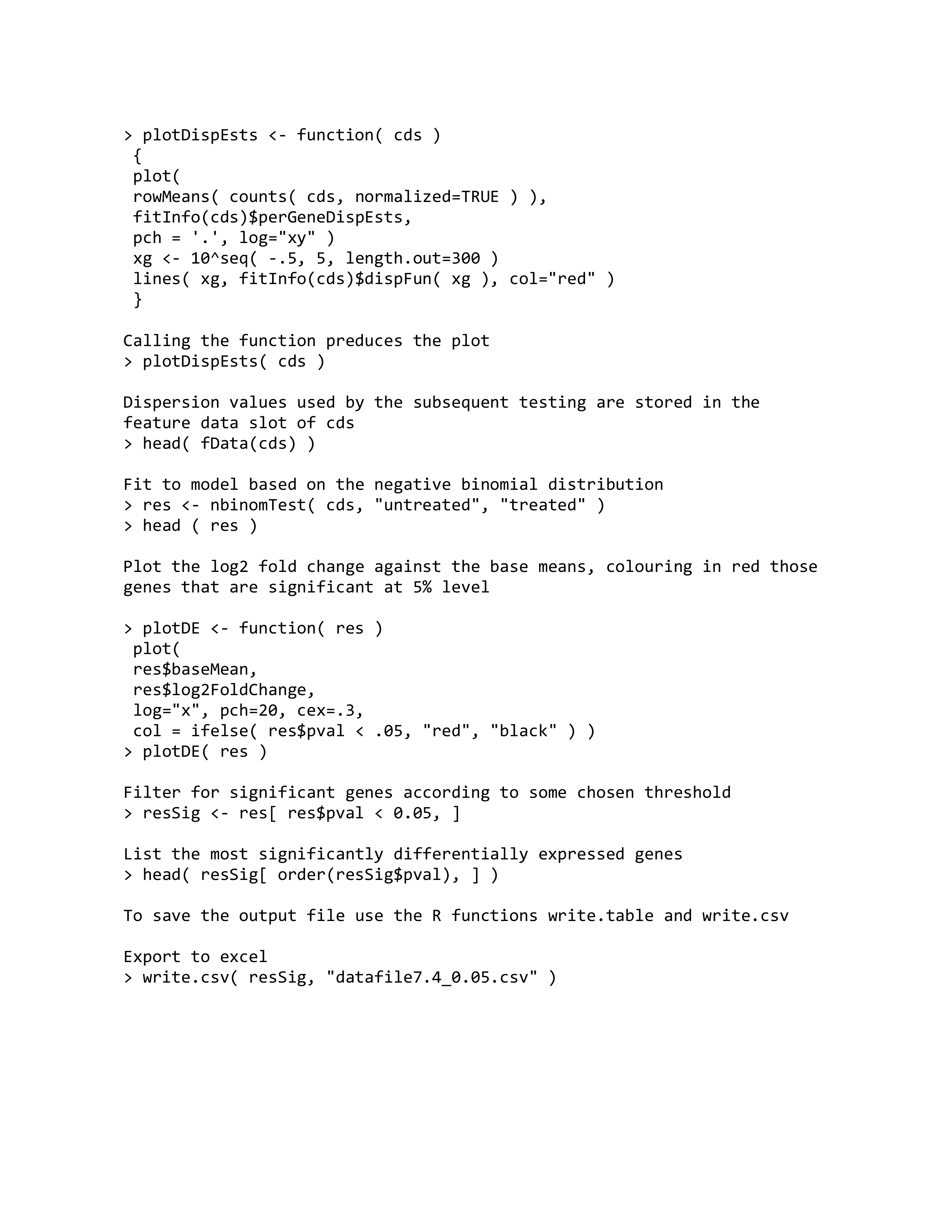

Supplement: Figure S1 — Commands and settings used in R for the statistical analysis. (TIF) [file pone.0089018.s001.tif]

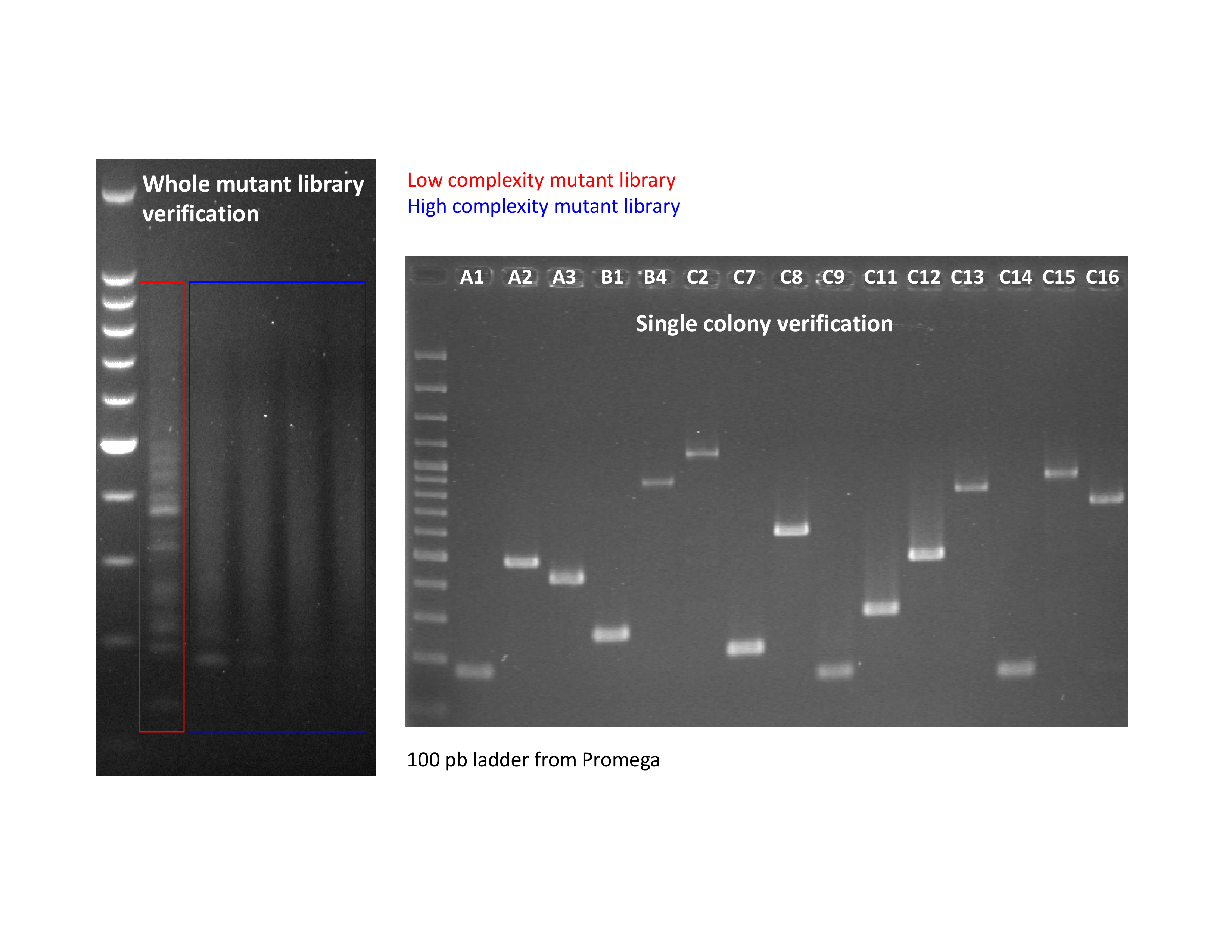

Supplement: Figure S2 — Whole mutant library and single colony verification. The gels show the result of the linker PCR used for library validation. The left gel shows squared in red a low complexity mutant library with a laddering of the smears. The blue squared lanes illustrate the same high complexity transposon mutant library from passage 0 (lane 2) to passage 3 (lane 5). The third generation transposon mutant library shows a smear with no specific bands. The right gel represents 15 randomly picked single mutant colonies isolated from the third generation transposon mutant library, each giving a band of different size indicating that the transposon has inserted at different locations with the genome. (TIF) [file pone.0089018.s002.tif]

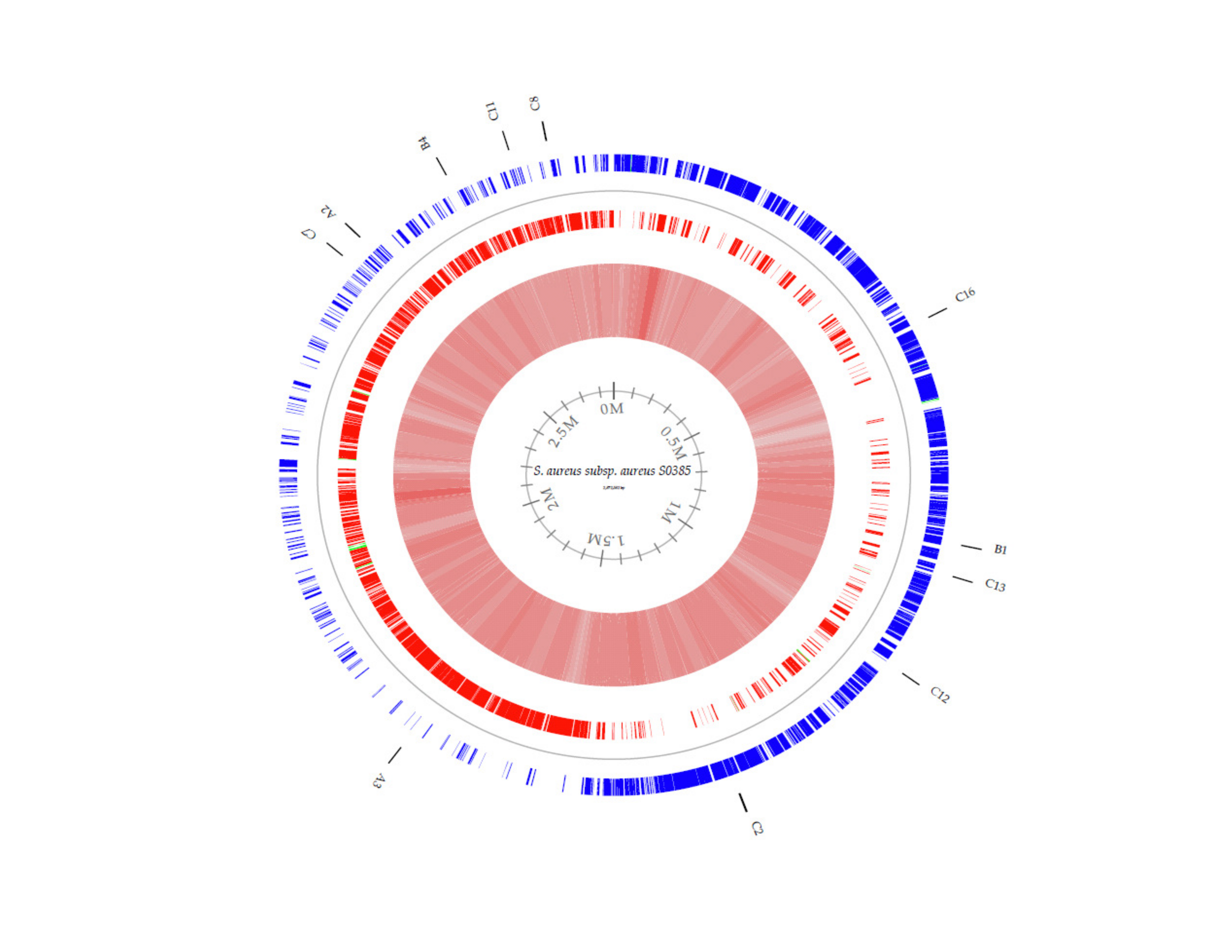

Supplement: Figure S3 — Genome atlas identifying transposon inserts of 11 random isolated mutants. The genome atlas illustrates by black marks in the outer most circle 11 different transposon insertion sites within the reference genome. The insertion sites were identified based on sequencing 11 of the 15 randomly picked mutant colonies described in figure S1. The fragments from the 11 mutants were sequenced and aligning to the reference genome. The blue and red parts of the atlas indicate forward and reverse transcriptional direction of the open reading frames within the reference genome. (TIF) [file pone.0089018.s003.tif]

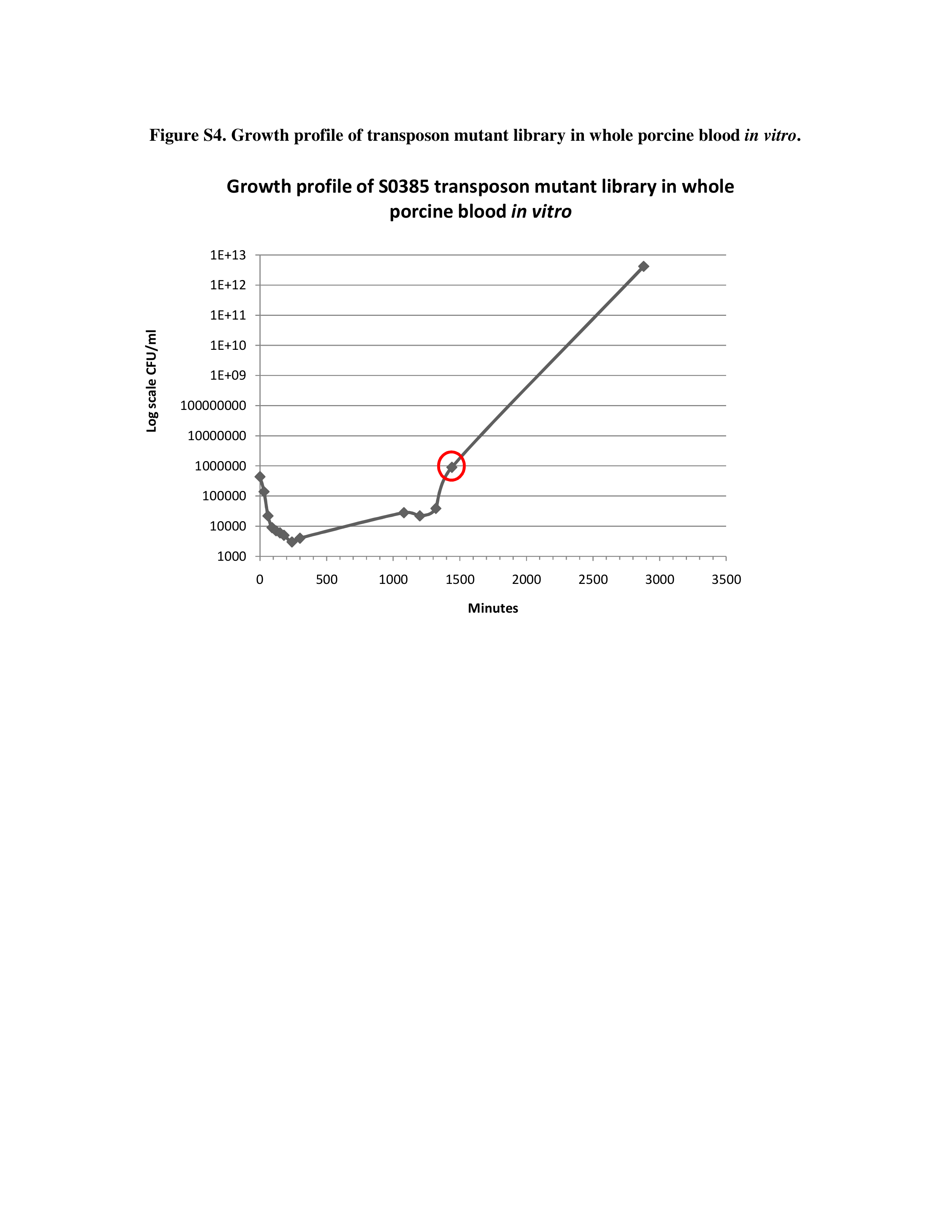

Supplement: Figure S4 — Growth profile of transposon mutant library in whole porcine blood in vitro . The figure shows the growth profile of the transposon mutant population in whole porcine blood in vitro. Mutant population size was determined at specific time-points to identify functionality of the blood immune cells. After 24 hours incubation in vitro the mutant population size was equivalent to the inoculated population size (indicated by the red circle). (TIF) [file pone.0089018.s004.tif]

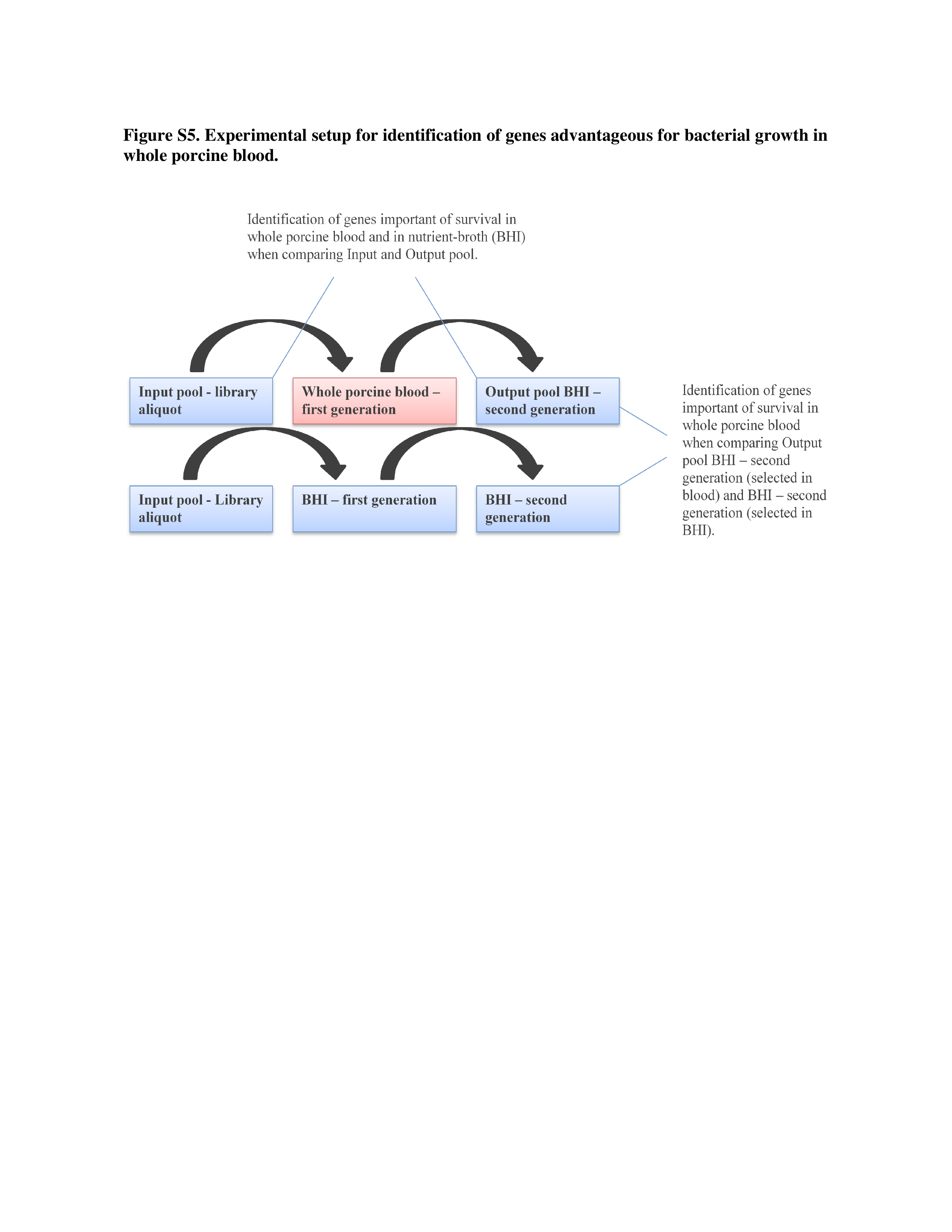

Supplement: Figure S5 — Experimental setup for identification of genes important for bacterial growth in whole porcine blood. The mutant composition in input pool pre-selection in whole porcine blood (Input pool - library aliquot) were compared with mutant composition in output pool post-selection in porcine blood (output pool BHI – second generation library). The mutants identified with a significant change in number of clones represent genes important for whole porcine blood survival in addition to growth BHI. The mutant composition in output pool post-selection in porcine blood (output pool BHI – second generation library) was compared to mutant composition after growth in BHI (BHI – second generation library). The mutants identified with a significant change in number of clones in both of the comparisons were evaluated as specific for survival in whole porcine blood in vitro. (TIF) [file pone.0089018.s005.tif]
